# Utilizing Kernel Density Estimation and Butterfly Diagram to Characterize the Gait Variability in the Fallers: A Cross‐Sectional Study

**DOI:** 10.1002/hsr2.70988

**Published:** 2025-06-30

**Authors:** Somayeh Mehrlatifan, Ali Fatahi, Davood Khezri

**Affiliations:** ^1^ Department of Sports Biomechanics, CT.C Islamic Azad University Tehran Iran; ^2^ Department of Sport Biomechanics and Technology Sport Sciences Research Institute Tehran Iran

**Keywords:** center of pressure, elderly, fall, gait, kernel density estimation, symmetry

## Abstract

**Background and Aims:**

The butterfly diagram is an effective tool for visualizing gait patterns and identifying potential areas of instability in the elderly individuals who fall. Nevertheless, there is a lack of comprehensive exploration regarding the quantification of variability at the intersections in butterfly diagrams. We proposed the utilization of kernel density estimation (KDE) and center of pressure (COP) symmetry index to analyze the spatial probability distribution of intersections in butterfly diagrams and to characterize the variability of gait patterns in elderly fallers.

**Methods:**

Twenty active elderly individuals (including both fallers and non‐fallers) volunteered to participate in this study. Initially, the self‐selected walking speed of each subject was assessed using a treadmill. Subsequently, each participant walked for a duration of 60 s. The bilateral toe‐off (TO) and initial contact (IC) points of the butterfly diagram were identified for the computation of the COP symmetry index and the intersections of bilateral TO‐IC. Following this, the intersections within the walking window were utilized to assess their density and variability through Kernel density estimation.

**Results:**

Fallers exhibited a significantly greater COP symmetry index (mean = 0.09, SD = 0.55), than non‐fallers (mean = 0.58, SD = 0.56; sig. = 0.03, η^2^ = 0.09). No significant differences were found in step width, step length, or COP distances (*p* > 0.05). KDE revealed distinct variability patterns: non‐fallers showed two patterns (A, B), while fallers displayed three (C, D, E), suggesting greater gait instability in fallers.

**Conclusions:**

KDE and COP symmetry analysis appeared to effectively quantify gait variability, offering insights into fall risk factors and potential intervention targets for elderly women.

## 1 Introduction

Gait variability and symmetry are commonly used as quantifiable measures of mobility in elderly individuals [1]. Muscle and functional imbalances heighten the risk of falls and instability while walking [2]. Although the connection between gait asymmetries and gait adaptation in older adults is widely debated, it is commonly believed that these imbalances can lead to falls and unstable walking patterns [3, 4]. Valid and reliable techniques to characterize variability in gait parameters are clinically important for assessing fall risks, or used as performance measures for evaluating responses to interventions, as well as for the design and follow‐up of rehabilitative programs [5, 6]. The trajectory of the center of pressure (COP) during walking is often depicted using a butterfly diagram. It has been reported that the butterfly diagram showcases fundamental gait features, including variability, stride width, and symmetry between the legs [7, 8].

Additionally, the butterfly diagram demonstrated a high level of variability in spatiotemporal parameters during both walking and running on a treadmill in adults [9]. In the laboratory setting, treadmill walking may produce a distinct pattern represented as an intersection in the butterfly diagram, identified by the point where two diagonal center of pressure (COP) shifts occur—from the initial contact (IC) to the subsequent opposite‐side toe‐off (TO)—reflecting the timing and spatial aspects of gait variability based on the positions of both feet [1]. The variability of gait has been linked to the standard deviations (SD) of the intersection points in both the anterior/posterior (AP) and medial/lateral (ML) directions [8]. Nevertheless, this method overlooked the connection between the intersection and foot positions, and may underestimate the effects of asymmetric gait patterns [1]. The individuals with multiple sclerosis (MS) who experienced falls showed greater variability in their step length and single support compared to those who did not fall [10, 11]. It has been revealed that in the butterfly diagram, the variability of the COP movement during walking, particularly in the AP direction, is linked to the degree of cerebellar impairment in individuals with multiple sclerosis [8].

Kernel density estimation (KDE) is a widely recognized statistical method commonly applied for estimating the probability density of data sets in geometric features, econometrics, and pattern recognition [12, 13, 14]. The utilization of KDE in point pattern analysis has been employed to assess the spatial spread of illnesses in the field of epidemiology [15, 16]. Furthermore, the results obtained from the KDE analysis were utilized for comparison with the traditional measures of gait symmetry in assessing the variability of walking after stroke [1]. In a similar manner, KDE can be utilized to ascertain the spatial probability distribution of intersections in the butterfly diagram. Up to now, the KDE method has not been utilized to assess gait performance and distinguish variability between individuals with and without a history of falls. Appling KDE to butterfly diagrams, the intersections can be likened to the events of interest, and the four points of bilateral IC and TO pertain to the specified area. Consequently, the KDE approach has the potential to uncover gait variability and offer quantitative values for a better comprehension of gait patterns [1].

Certain temporal gait parameters exhibited stronger correlations with the risk of falls during comfortable single‐task walking. and spatial gait variables contributed significantly to the classification model [17]. Gait variables appear to be sensitive indicators of gait impairment and identify the risk of falling [18, 19, 20, 21, 22, 23, 24, 25]. The elderly are at a higher risk of injury and mortality due to falls, which can impact their quality of life, and their fear of falling again reduces their activation. This reduced mobility can increase the risk of falls. Hence, promoting mobility is crucial for healthy aging in this population [26, 27, 28, 29]. Gait constitutes a significant risk factor for falls among older adults, underscoring the importance of identifying the determinants contributing to fall risk [30, 31]. This study aimed to investigate the variety of walking in fallers and non fallers elderly by applying KDE in intersections and COP direction. We used KDE analysis to investigate properties of spatial probability distributions in intersections in a butterfly diagram and compare the obtained measures with conventional measures of gait symmetry and variability between elderly fallers and nonfallers.

## 2 Methods

This study conducted a secondary analysis of our previous cross‐sectional study conducted by Mehrlatifan S, Fatahi A, and Khezri D in 2023, titled “Frequency Content of GRF during Walking: Comparison in Elderly Fallers and Non‐Fallers” [31]. In fact, we only requested the subjects to participate in other tests.

### 2.1 Participants

Twenty senior women who were willing to participate in the study were selected using an convenience sampling method. The inclusion criteria were established as follows: Participants must have experienced at least one documented fall within the preceding twelve months, aligning with the study's emphasis on fallers; they must be adults aged 65 years or older, reflecting the frequent focus on gait variability and falls in older populations where such issues predominate; they should be able to walk independently, either with or without assistive devices such as canes or walkers, to facilitate gait analysis on a treadmill or comparable equipment; they must be willing and capable of providing informed consent for participation; and they need to complete gait assessment procedures to produce data suitable for analysis via Kernel Density Estimation and butterfly diagrams. The exclusion criteria were outlined as follows: Individuals with conditions that might hinder their ability to comprehend and follow gait testing instructions; those with recent injuries (e.g., fractures within the prior 6 months) or acute health issues (e.g., stroke or infection) that could transiently affect gait patterns unrelated to chronic variability; those with disorders such as severe Parkinson's disease or multiple sclerosis, unless explicitly included in the study design, due to their potential to obscure gait variability not directly linked to fall risk; individuals incapable of walking or requiring complete assistance, which would preclude effective gait data acquisition; and participants who cannot finish the gait assessment or whose data is inadequate for analysis with the designated methods (e.g., due to technical issues or unwillingness to complete the protocol). The participants were divided into two groups, one with a history of falling and the other without, with 11 participants in the fallers and 9 in the nonfallers group. Table 1 shows the demographic information of the participants and the results of an independent t‐test for body mass (*p* = 0.518) and height (*p* = 0.863) variables, which showed no significant difference existed between both Groups. The study was ethically approved by the Sport Sciences Research Institute of Iran under the ethics SSRI. REC‐2212‐1977.

**Table 1:** Demographic information of the participants and results of independent *t*‐test.

Variable	Fallers	Non‐Fallers	*t*	*p* value
Body Mass (kg)	65.73 ± 12.47	62.45 ± 10.80	0.658	0.518
Height (cm)	160.05 ± 7.51	159.10 ± 11.58	0.175	0.863
Age (year)	69 ± 4.5	65 ± 4.9	0.115	0.724

### 2.2 Experimental Protocol

**Figure 1:**
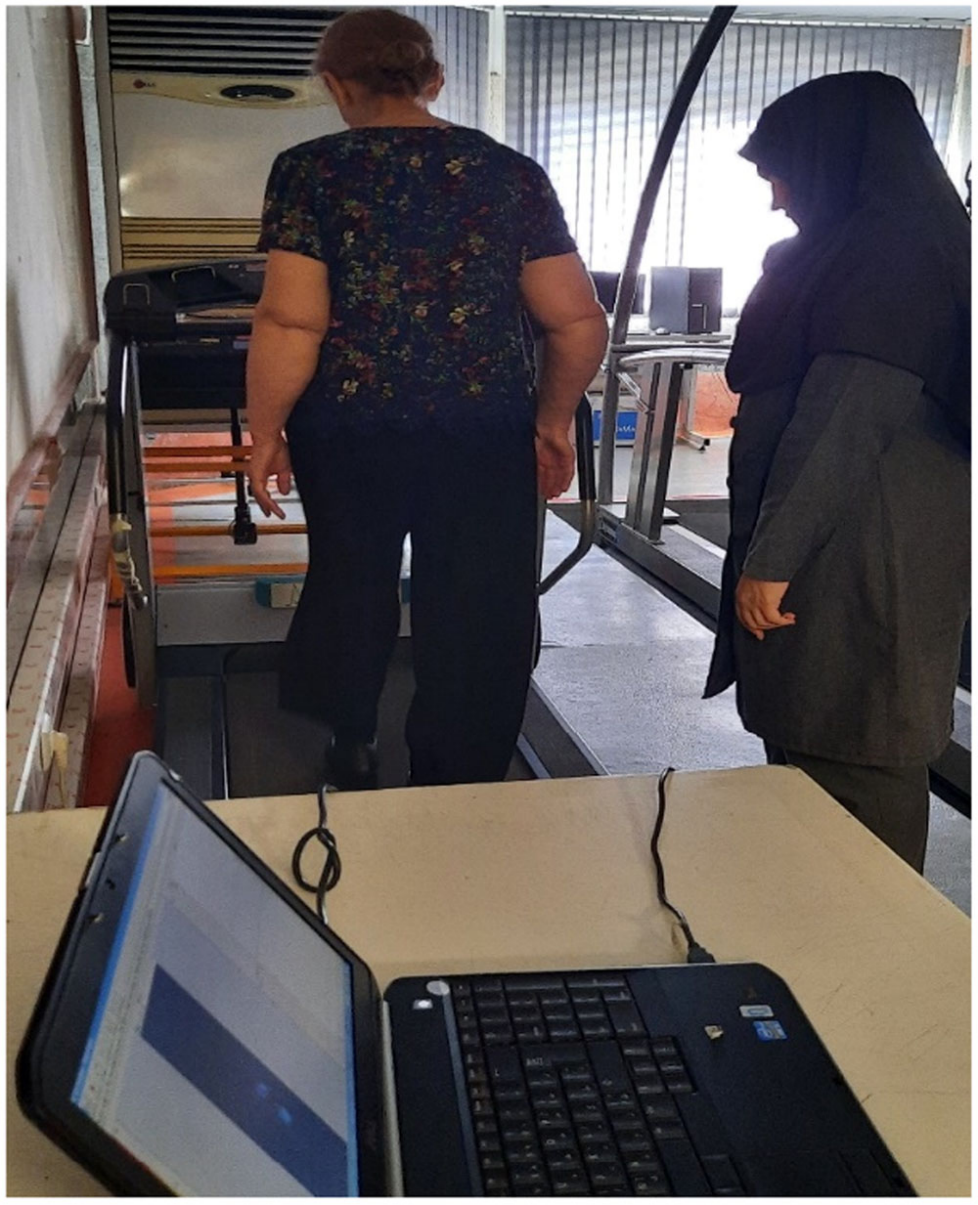
Gait lab during the test.

Participants ambulated on a treadmill equipped with force plates (Zebris Medical GmbH‐ Germany ‐ Gait analysis FDM‐T). Initially, we assessed the self‐selected walking speed of each participant using the treadmill based on their previous experience. After determining their walking speed, each participant walked for a duration of 60 s. The temporal and spatial components of gait variability and COP trajectories were recorded at a sampling rate of 1000 Hz [1, 32]. In Figure 1, the subjects are shown during the test.

### 2.3 Data Processing

**Figure 2:**
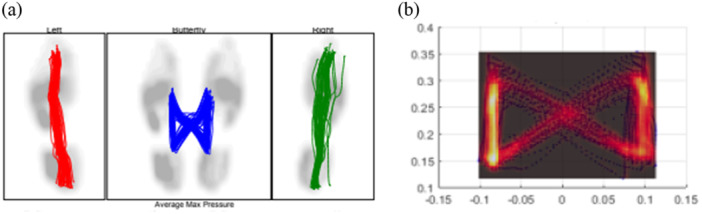
(a) Butterfly diagram, The left box shows the COP direction in the left leg, The right box shows the COP direction in the right leg, and the middle box shows moving of COP direction between the two legs. (b) kernel density estimation diagram of one of the participants of the control group. The walking COP trajectory formed a “butterfly” graphic pattern. A non‐fallers individual's typical butterfly diagram (Figure 2a) and density distribution (Figure 2b) are depicted in Figure 2. The density distribution plot provides four pieces of information. First, the black box in Figure 2b corresponds to the area of the COP trajectory, which is the area of the butterfly diagram in Figure 2a. Second, the yellow region represents the highest density of intersection distribution and the location of COP trajectory area. Third, the red area immediately external to the yellow region represents how other intersections deviated from the point of highest density. Fourth, the vertical oval shape indicated that the distribution of intersections was mainly in the AP direction, versus the ML direction.

The collected data on COP were analyzed offline using custom “MATLAB, version R2021B (Mathworks, Natick, MA, USA)”, filtered with Buterwoth 4th order filter, 10 Hz cutoff frequency [1]. The butterfly diagram and kernel density estimation diagram are shown in Figure 2a,b. COP trajectory during walking was generated as a graphic pattern of “butterfly” (Figure 2b). The COP symmetry index was calculated using the following equation [1]:

COP Symmetry Index=(LCOP length−RCOP length)/(LCOP length+RCOP length)

**Figure 3:**
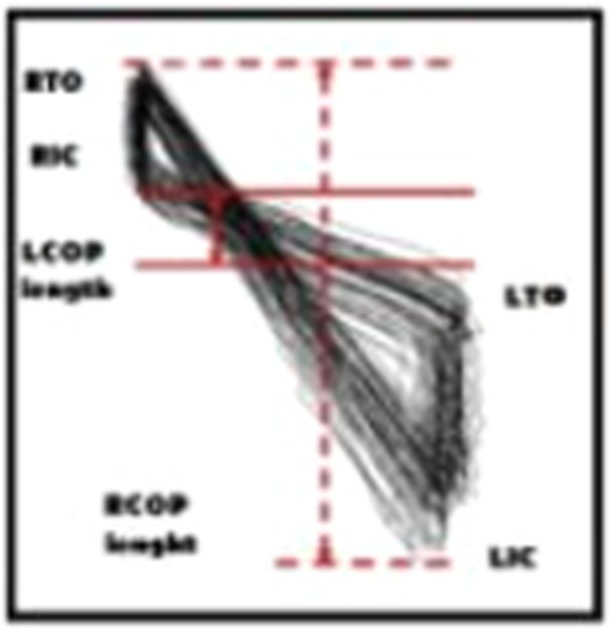
Four key points of left and right initial contact (IC) and toe off (TO) were determined. LCOP; left COP and RCOP; right COP. LTO; left TO and RTO; right TO [1].

Four key points of left and right initial contact (IC) and toe off (TO) were determined using 20 N threshold based on vertical component of the ground reaction force data (Figure 3) [1]. where left COP (L COP) and right COP (R COP) lengths were calculated as the absolute AP distance. The left COP length was between left TO (LTO) and consecutive right IC (RIC), while right COP length was between right TO (RTO) and consecutive left IC (LIC). In addition, the distances from LTO to RIC and RTO to LIC as well as the intersection between two lines of LTO‐RIC and RTO‐LIC were determined. The mean of step width, left step length, and right length were also derived directly from the treadmill output. To assess variability, the kde2d function from Botev et al. [33] was employed to apply and calculate the kernel density estimation (KDE) in MATLAB. The kde2d function was employed with the inputs of coordinates, gait cycle numbers, and maximum AP and ML range. To eliminate the effects of acceleration and deceleration in walking, the first and last three steps of each trial were excluded [1]. Typically, for the number of gait cycles average of 50 intersections were used for the calculations. The computational area was defined by the maximum and minimum values of toe‐off (TO) and initial contact (IC) for each participant. We then calculated the maximum density and compared it across different groups.

### 2.4 Statistic Analysis

Shapiro‐wilk test presented normal distribution of the data. For statistical comparisons, all parameters were averaged across all gait cycles for each participant. Independent *t*‐tests (2 ‐tailed) were employed to compare the COP symmetry index, the distances of RTO‐LIC and LTO‐RIC, highest density values, and the step width, step length, between the two groups of fallers and nonfallers. Statistical significance was determined for *p*‐values less than or equal to 0.05.

## 3 Results

The statistical analysis of gait parameters is summarized in Table 2, which presents the mean and standard deviation (SD) for step width, step length (left and right), distances, center of pressure (COP) symmetry index, and kernel density estimation (KDE) for both faller (F) and non‐faller (nF) Groups.

**Table 2:** Summary of gait parameters across participant groups.

Statistics	Step width (cm)	Step length L (cm)	Step length R (cm)	Distance 1 (cm)	Distance 2 (cm)	COP symmetry index	KDE
Mean F	8.18	34.29	33.68	132.88	138.27	0.09	654.57
SD F	3.75	10.21	10.59	102.04	100.74	0.55	616.03
Mean nF	12.22	36.37	37.67	228.75	201.69	0.58	462.42
SD nF	6.79	5.57	5.89	182.36	189.50	0.56	312.43
*t*	−1.69	−0.55	−1.01	−1.07	−0.96	1.23	0.85
Sig	0.13	0.59	0.33	0.29	0.35	0.03^a^	0.40
ES (η^2^) (interpretation)	0.13 (medium)	0.02 (small)	0.05 (small)	0.06 (small)	0.04 (small)	0.09 (medium)	0.03 (small)

Abbreviations: Distance1, distance of LTO‐RIC; Distance2, distance of RTO‐LIC; df, degrees of freedom; F, fallers group; L, left foot; nF, non fallers group; R, right foot.

^a^ significant difference

The mean step width for faller Group was 8.18 ± 3.75 cm, lower than the non‐faller Group, which presented 12.22 ± 6.79 cm, revealed no significant difference with medium effect size (*p* = 0.13, η^2^ = 0.13). The mean step lengths for faller were 34.29 ± 10.21 cm (left) and 33.68 ± 10.59 cm (right), while the non‐faller Group exhibited greater step lengths of 36.37 ± 5.57 cm (left) and 37.67 ± 5.89 cm (right), indicating a potential difference in stride efficiency with small effect size in comparion (*p* = 0.59, η^2^ = 0.02) and (*p* = 0.33, η^2^ = 0.05) for left and right, respectively.

Distance Comparion did not revealed significant differences, with faller Group averaging 132.88 ± 102.04 cm (Distance 1) and 138.27 ± 100.74 cm (Distance 2), compared to 228.75 ± 183.36 cm and 201.69 ± 189.50 cm for the non‐faller Group, presenting small effect size, (0.06 and 0.04), respectively. The COP symmetry index showed a significant difference (sig.=0.03, η^2^ = 0.09), with the non‐faller Group demonstrating a greater mean of 0.58 ± 0.56 cm compared to faller 0.09 ± 0.55 cm, indicating better balance and stability in the non‐faller Group.

Effect sizes (η²) were calculated to assess the practical significance of these findings. The COP symmetry index exhibited a medium effect size (0.13), while other parameters showed small effect sizes, suggesting varying degrees of relevance across the measured variables. These results underscore the importance of considering gender differences in gait analysis and their implications for rehabilitation and clinical practices.

**Figure 4:**
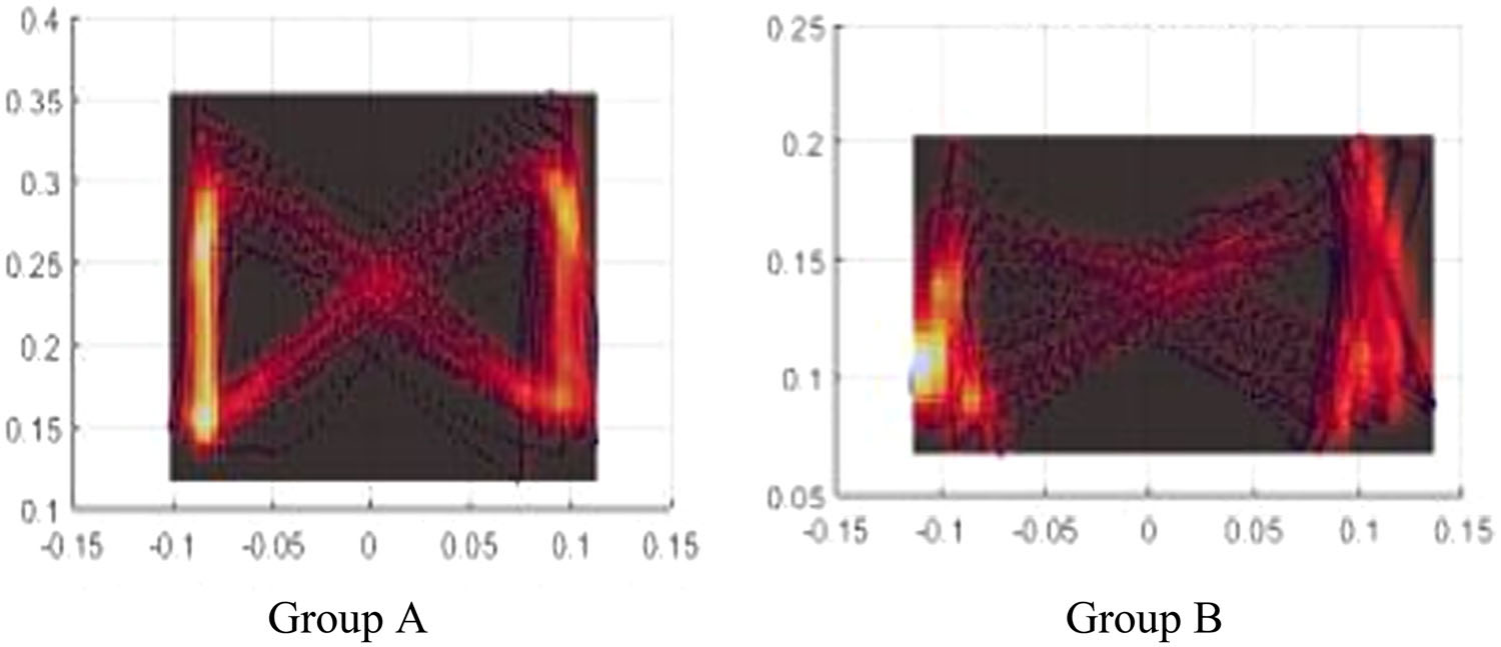
Ggroup A, which showed the type A pattern, included 5 non‐faller participants and Group B, which showed the type B pattern, included 4 non‐faller participants.

**Figure 5:**
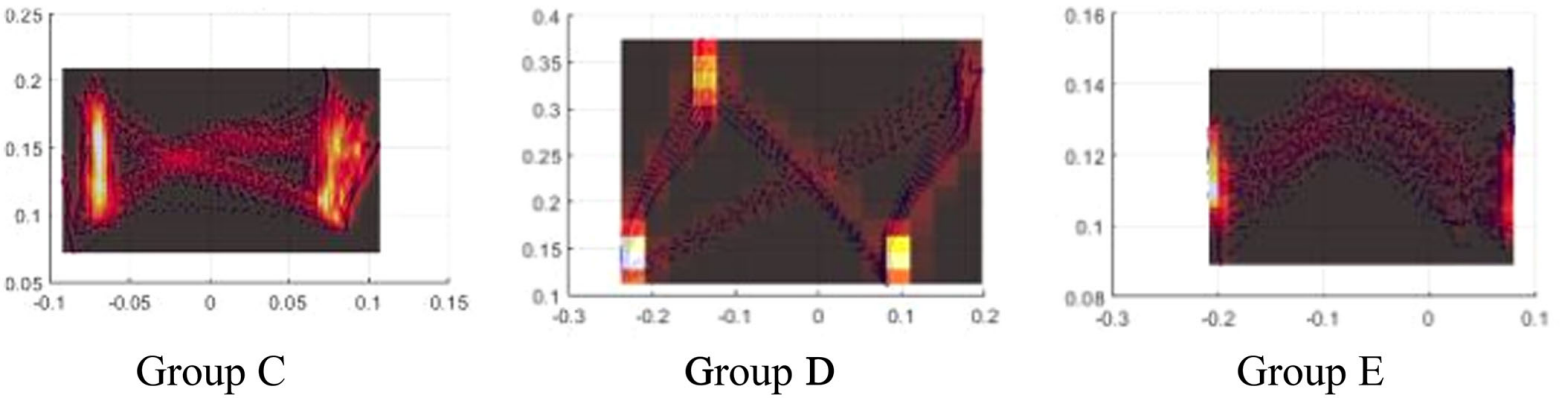
Group C, which showed the pattern type C, included 5 faller participants, Ggroup D, which showed the pattern type D, included 4 faller participants and Group E, which showed the pattern type E, included 2 faller participants.

We found two different types of density distributions in the nonfaller participants which were named Group A (*n* = 5) and Group B (*n* = 4) and three types in the faller that were named Group C (*n* = 5), Group D (*n* = 4) and Group E (*n* = 2). Which are shown in the Figures 4 and 5.

In Figure 4, it is evident that pattern type A exhibited Almost a similarity in the range of both AP and ML, with the intersection being shifted around the center. And, in type B it seemed to show the same pattern as Group A, but the main difference was observed in larger ML range comparing to AP range.

In Figure 5, it is apparent that pattern type C displayed a variation in center of pressure (COP) density in the anteroposterior (AP) direction, with the intersection shifting away from the center and aligning more closely with the left foot, indicating AP variation. Additionally, the variability in the direction of COP is observable between the toe‐off (TO) and initial contact (IC) phases. In pattern type D, three peaks of COP density are noted along the diagonal trajectory of the COP, in contrast to the previously observed type. For pattern type E, the COP density in the AP direction was nearly uniform on both sides; however, the distinction lies in the positioning of the intersections, which were situated beyond the space between the feet and somewhat outside the AP range.

## 4 Discussion

Falls in older adults constitute a pressing public health concern; however, the existing body of research exhibits a notable deficiency in thoroughly delineating the gait variability that heightens susceptibility to such events. Conventional approaches to gait analysis frequently depend on rudimentary parameters, such as stride length or walking speed, which inadequately reflect the intricate, oscillatory dynamics of instability—most notably the nuanced asymmetries or shifts in the center of pressure (COP) that differentiate fallers from their non‐falling counterparts. This shortfall impedes the formulation of accurate fall risk evaluations and the design of specific, effective interventions. The impetus for this investigation stemed from the critical need for sophisticated analytical methodologies capable of both quantifying and visually depicting these multifaceted gait anomalies, thereby advancing insight into the mechanisms underlying falls and supporting the development of preventative measures. To rectify this deficiency, the current study utilized kernel density estimation (KDE) and butterfly diagrams to examine gait variability among elderly fallers, presenting an innovative framework that reconciles statistical precision with practical utility in gait research.

This study employed a novel method to assess the comparable COP symmetry index and distances between individuals who experienced falls and those who did not. We used the observation method in the KDE analysis and successfully determined the spatial distribution and configuration of intersections for fallers while walking in the butterfly diagram, distinguishing them from non‐fallers. Additionally, distinctive spatial patterns were identified within both the falling and non‐falling groups.

The COP symmetry index is an index used in biomechanics to assess the balance and weight distribution between the left and right sides of the body during gait. Our results suggest that this index is notably elevated in individuals who fall compared to those who do not. This indicates that the variability in the center of pressure (COP) between the left and right feet is greater among fallers. The asymmetry observed may indicate muscular and functional imbalance. The subject of gait imbalances and their connection to gait adjustment in elderly individuals is a topic of extensive discussion. It is generally thought that muscular and functional imbalances may elevate the likelihood of falls [34] and contribute to gait instabilities [35, 36]. While the COP symmetry index is capable of distinguishing between elderly fallers and non‐fallers, in the present statement, we emphasize the significance of exercising caution while approaching the aforementioned conclusion, as the crucial elements of movement production and control may exhibit variations. Thus we underscore the need for careful consideration and evaluation of the factors that influence movement production and control at the same time as conducting such experiments.

The results did not reveal any statistically significant difference in distance 1 and distance 2, step length, and step width between the two groups. Hence, it appears that older individuals with a prior history of falls may employ compensatory mechanisms to uphold balance and stability during walking [30]. Furthermore, there is a possibility suggesting that these variables lack validity in differentiating between the diagnosis of falls in elderly individuals.

While no statistically significant difference was identified in terms of width step, length step and distance, some distinctions were observed through visual comparison. We observed two distinct density distribution patterns among nonfallers, designated as Groups A and B. Group A exhibited comparable ranges in both the anterior‐posterior (AP) and medial‐lateral (ML) directions, while Group B displayed a larger ML range than AP. Group A comprised 5 non‐faller participants, and Group B comprised 4 non‐faller participants. It appears that non‐faller elderly individuals demonstrated a consistent gait pattern, despite the identification of two distinct groups. This suggests that healthy and active elderly individuals without a history of falling may exhibit predominantly one of the two patterns, A or B.

Also this study identified three distinct density distribution patterns among fallers: Groups C, D, and E. Group C showed differences in COP density in AP, with the intersection moved away from the center and closer to the left foot, indicating variability in COP's direction between TOs and ICs. Group D exhibited three points of COP density peak along the diagonalization of the COP trajectory, while Group E showed nearly equal COP density in AP on both sides, with intersections located beyond the space between feet and out of the AP range. Group C comprised 5 faller participants, Group D comprised 4, and Group E comprised 2 faller participants. In the analysis of gait patterns among elderly individuals prone to falling, there is clear evidence of asymmetry in the anteroposterior (AP) or mediolateral (ML) planes, or a displacement of the center of pressure (COP) beyond the range of the lower limbs. This observed instability in gait dynamics is likely a contributing factor to the incidence of falls in this population. Consequently, these findings imply that gait variability in elderly fallers may exceed that observed in their non‐falling counterparts.

It has been reported that the variability of COP in the butterfly diagram could be beneficial in identifying runners who are at a higher risk of lower limb injury [37]. The butterfly diagram has also differentiated the walking characteristics of individuals with multiple sclerosis (MS) [10, 11] and those without it [8]. We also noticed resemblances between Group B of non‐fallers and Group C of fallers. It seems that people who are in Group B may be at risk of falling. Certainly, if this assertion holds true, it offers a valuable evaluation of the assessment and estimation of the risk of falling in the elderly and warrants further extensive research for in‐depth investigation.

### 4.1 Limitation

Although the use of kernel density estimation (KDE) and butterfly diagrams provides an innovative method for assessing gait variability in fallers, certain limitations should be recognized. Initially, the limited sample size of both fallers and non‐fallers, along with the inclusion of female participants in the study, may restrict the generalizability of the findings to a wider population. Expanding the cohort to include a wider range of ages, health statuses, and environmental conditions could improve the validity of the findings. Secondly, focusing solely on specific gait metrics—such as center of pressure (COP) displacement and asymmetry in the anteroposterior (AP) or mediolateral (ML) directions—might neglect other critical biomechanical or neuromuscular elements influencing fall occurrences. Thirdly, the study's observational design hampers the establishment of a direct causal link between gait variability and fall risk, given that potential confounding factors (e.g., medication effects, cognitive decline, or footwear variations) were not comprehensively accounted for. Moreover, the use of KDE presupposes a continuous gait data distribution, potentially failing to capture discrete events or outliers that may affect variability trends. Lastly, while the butterfly diagram serves as a useful visual aid, its qualitative nature may lead to subjective interpretations, possibly compromising its consistency across diverse research contexts. To overcome these shortcomings, future research could adopt longitudinal approaches, incorporate a wider array of gait parameters, and implement standardized validation procedures to enhance the dependability and utility of the proposed techniques.

## 5 Conclusions

This study employed kernel density estimation (KDE) and butterfly diagrams to characterize gait variability, revealing significantly increased oscillatory patterns in the gait of elderly fallers compared to non‐fallers. These findings justify the investigation by demonstrating that such analytical tools can effectively quantify and visualize gait irregularities, offering a clearer understanding of the biomechanical underpinnings of falls in this population. By identifying increased variability in discrete parameters, such as center of pressure (COP) displacement, this study advances the current state of knowledge beyond traditional gait analysis methods, which often rely on simpler metrics or qualitative assessments. The integration of KDE provides a statistically robust representation of gait variability distributions, while the butterfly diagram offers an intuitive visual tool to discern asymmetries and instabilities, enhancing the precision of fall risk evaluation.

These advancements hold practical implications for developing targeted rehabilitation strategies and fall prevention programs. For instance, the observed changes in COP variability, as emphasized through butterfly diagrams, could serve as a reliable indicator for evaluators to assess fall risk and design personalized interventions, potentially reducing fall‐related injuries and associated healthcare costs. This approach not only refines our comprehension of gait dynamics but also lays the groundwork for its application in broader contexts, such as improving athletic performance or monitoring motor rehabilitation outcomes.

To further strengthen these insights, future experiments should explore longitudinal designs to track gait variability over time and establish causal links between COP fluctuations and fall incidents. Additionally, incorporating a wider range of biomechanical and environmental variables—such as muscle activation patterns, terrain differences, or footwear effects—into the KDE analysis could provide a more comprehensive fall risk profile. Validation studies comparing butterfly diagram interpretations across diverse populations and settings are also recommended to ensure their consistency and utility. Collectively, these efforts could solidify the role of KDE and butterfly diagrams as standardized tools in gait research, advancing both clinical practice and scientific inquiry into fall prevention.

## Author Contributions

**Somayeh Mehrlatifan:** conceptualization, formal analysis, software. **Ali Fatahi:** conceptualization, formal analysis, supervision, software. **Davood Khezri:** conceptualization, data curation, formal analysis, software.

## Ethics Statement

All authors have read and approved the final manuscript. A.F. had full access to all data and takes responsibility for its integrity and analysis accuracy.

## Consent

Informed consent was obtained from all subjects involved in the study.

## Conflicts of Interest

The authors declare no conflicts of interest.

## Transparency Statement

The lead author Ali Fatahi affirms that this manuscript is an honest, accurate, and transparent account of the study being reported; that no important aspects of the study have been omitted; and that any discrepancies from the study as planned (and, if relevant, registered) have been explained.

## Data Availability

The data presented in this study are available on request from the corresponding author.
